# Sphingosine‐1‐phosphate suppresses chondrosarcoma metastasis by upregulation of tissue inhibitor of metalloproteinase 3 through suppressing miR‐101 expression

**DOI:** 10.1002/1878-0261.12106

**Published:** 2017-08-08

**Authors:** Chun‐Hao Tsai, Dong‐Ying Yang, Chih‐Yang Lin, Tsung‐Ming Chen, Chih‐Hsin Tang, Yuan‐Li Huang

**Affiliations:** ^1^ School of Medicine China Medical University Taichung Taiwan; ^2^ Department of Orthopedic Surgery China Medical University Hospital Taichung Taiwan; ^3^ Graduate Institute of Basic Medical Science China Medical University Taichung Taiwan; ^4^ Department of Pharmacology China Medical University Taichung Taiwan; ^5^ Department and Graduate Institute of Aquaculture National Kaohsiung Marine University Kaohsiung Taiwan; ^6^ Department of Biotechnology College of Medical and Health Science Asia University Taichung Taiwan; ^7^ Department of Medical Research China Medical University Hospital Taichung Taiwan

**Keywords:** chondrosarcoma, metastasis, microRNA, sphingosine‐1‐phosphate, tissue inhibitor of metalloproteinase

## Abstract

Chondrosarcoma is the second most common primary malignancy form of bone cancer, exhibiting resistance to chemotherapy and radiation therapy as well as developing high metastasis ability in late‐stage tumors. Thus, understanding the metastatic processes of chondrosarcoma is considered a strategy for the treatment of this disease. Sphingosine 1‐phosphate (S1P), a bioactive sphingolipid, is produced intracellularly by sphingosine kinase (SphK) and is regarded as a second signaling molecule that regulates inflammation, proliferation, angiogenesis, and metastasis. However, the effect of S1P on chondrosarcoma remains uncertain. As demonstrated by the transwell, immunoblotting, and real‐time PCR analyses, we found that S1P inhibited cell migration and MMP‐2 expression through the upregulation of the tissue inhibitor of metalloproteinase‐3 (TIMP‐3) expression in human chondrosarcoma cells. Additionally, we also showed that microRNA (miRNA)‐101, which targets the 3′ untranslated region (3′UTR) of TIMP‐3, decreased significantly following S1P treatment. After transfection with miR‐101 mimics, the S1P‐regulated cell migration and TIMP‐3 expression were both reversed. Furthermore, we also showed that the S1P‐inhibited cell migration is mediated through the c‐Src/MEK/ERK signaling axis. Meanwhile, the *in vivo* study indicated that overexpression of SphK1 decreases chondrosarcoma metastasis to the lungs. Our results illustrate the clinical significance between SphK1, TIMP‐3, and miR‐101 in human chondrosarcoma patients. Taken together, our results suggest that S1P and miR‐101 may prove to be potential therapeutic targets for future chondrosarcoma treatment.

**Abbreviations**
3′UTR: 3′ untranslated region
microRNA: miRNA
S1P: sphingosine‐1‐phosphate
siRNA: small interfering RNA
SphK: sphingosine kinase
TIMP: tissue inhibitor of metalloproteinase

## 1 Introduction

Chondrosarcoma, a cartilage‐forming neoplasm, belongs to a member of tumors of bone and soft tissue known as sarcomas. It is the second most common bone malignancy and occurs in about 20–27% of all bone cancer cases (David *et al*., 2011). Unlike other bone cancers, it mainly affects adults and is relatively rare in children and teenagers (Leddy and Holmes, 2014). Based on tumor sizes, cellularity, and nuclear staining, chondrosarcoma can categorize into at least three histological grades and high‐grade tumors predictably possessing the worse outcomes. Until now, the histological grade is still the best indicator of prognosis in chondrosarcoma (Schwab *et al*., 2012). Chondrosarcoma is resistant to both chemotherapy and radiation; thus, surgical excision forms the popular option of current chondrosarcoma treatment (Gelderblom *et al*., 2008).

Metastasis, a complex process in which tumor cells spread out from one part of body to another, has been a major clinical problem causing approximately 90% of cancer patient mortality. The breakdown of the basement membrane surrounding the tumor cells and also the increase in the abilities of proliferation, migration, and invasion are the hallmarks of metastasis (Deng *et al*., 2014). The well‐studied proteases linked with the breakdown of the extracellular matrix (ECM) surrounding the tumor cells are the matrix metalloproteinases (MMPs) (John and Tuszynski, 2001). Several clinical and experimental studies have demonstrated that elevated levels of MMPs are associated with cancer progression and responsible for shortening patient survival (Yang *et al*., 2014). MMP‐1, ‐2, ‐3, ‐9, and ‐13 are expressed in human chondrosarcoma cells (Gebauer *et al*., 2005). Among these MMPs, MMP‐2 plays a crucial role in cancer metastasis by the degradation of type IV collagen, the major component of the cartilage (Roomi *et al*., 2013). Furthermore, inhibition of MMP‐2 suppresses migration and metastasis of chondrosarcoma (Power *et al*., 2013; Tsai *et al*., 2015). Thus, manipulation of MMP‐2 may regulate the chondrosarcoma metastasis. The activity of MMPs can be regulated by their endogenous inhibitors, called the tissue inhibitor of metalloproteinases (TIMPs). Until now, four members of TIMPs were identified, named TIMP‐1–4, and all of them are capable of inhibiting MMPs (Arpino *et al*., 2015). Unlike TIMP‐1, ‐2, and ‐4 which are regarded to be soluble, TIMP‐3 is ECM bound and not only inhibits the activity of MMPs, but also suppresses ADAMs (A Disintegrin And Metalloproteases) that are not inhibited by other TIMPs (Yu *et al*., 2000). Furthermore, knockdown of TIMP‐3 enhances tumor growth, angiogenesis, and invasion (Cruz‐Munoz *et al*., 2006; Zhang *et al*., 2010).

Sphingosine 1‐phosphate (S1P) is generated intracellularly through the series enzymatic metabolisms of sphingolipids. Among them, the sphingosine kinases (SphKs) are the major enzymes responsible for the production of S1P from its precursor, sphingosine. Two distinct isoforms of SphKs were identified, termed SphK1 and SphK2. Both of them possess different biochemical properties, subcellular distribution, and physiological roles (Hannun and Obeid, 2008). SphK1 is overexpressed in multiple types of cancers, which appears to contribute to carcinogenesis, chemo‐ or radioresistance, and poor prognosis. The roles of SphK‐2 in cancer, however, are not well characterized (Pyne *et al*., 2016). S1P can either be exported through the membranous specific transporter proteins and act as an extracellular first messenger ligand for its specific G protein‐coupled receptors (S1P_1–5_) (Blaho and Hla, 2014) or can serve as an intracellular second messenger to bind to the specific intracellular target proteins (Hait *et al*., 2009). In turn, SphK1 and S1P have been shown to promote tumor growth, tumor angiogenesis, metastasis, and resistance to apoptosis in several cancers (Huang *et al*., 2011). However, a recent study indicated that S1P inhibits migration through the activation of S1P_2_ and ROCK‐mediated vimentin S71 phosphorylation in human breast cancer and anaplastic thyroid cancer cells (Hyder *et al*., 2015). Additionally, S1P inhibits endothelial cell angiogenesis as a result of the TIMP‐2 induction from vascular smooth muscle cells (Mascall *et al*., 2012). Furthermore, several studies showed the evidence that S1P suppresses cell proliferation through the inactivation of Akt in keratinocytes (Kim *et al*., 2004) and prostate cancer cells (Chang *et al*., 2009; Huang *et al*., 2014). Thus, the functions of S1P on cancer cells remain controversial, especially because there is no study on chondrosarcoma. Herein, we showed for the first time that S1P inhibits human chondrosarcoma migration through the induction of TIMP‐3 by downregulation of microRNA‐101 (miR‐101).

## 2 Materials and methods

### 2.1 Ethics statement

All animal work was conducted in accordance with a protocol approved by the China Medical University (Taichung, Taiwan) Institutional Animal Care and Use Committees (IACUC Approval No. 2016‐155 to CHT). Regarding the collection of clinical samples, the study protocol was approved by the Institutional Review Board of China Medical University Hospital. All patients gave written consent before enrollment. Tumor tissue specimens were collected from patients diagnosed with chondrosarcoma who underwent surgical resection at China Medical University Hospital.

### 2.2 Reagents

d‐Erythro‐sphingosine 1‐phosphate (S1P) was purchased from Avanti Polar Lipid Inc. (Alabaster, AL, USA). The anti‐rabbit sphingosine kinase 1, a tissue inhibitor of metalloproteinase 3, was obtained from Genetex (Irvine, CA, USA); anti‐rabbit p‐c‐Src and p‐MEK were obtained from Cell Signaling Technology (Danvers, MA, USA); anti‐rabbit p‐ERK, ERK, MEK, MMP‐2, and c‐Src were obtained from Santa Cruz (Santa Cruz, CA, USA). The inhibitors for Src (PP2) and MEK (PD98059 and U0126) were purchased from Sigma‐Aldrich (St. Louis, MO, USA). The Luciferase assay kit was purchased from Promega (Madison, WI, USA). Sphingosine kinase‐1 cDNA clone plasmids were purchased from OriGene (Rockville, MD, USA). miR‐101 mimic was purchased from Invitrogen (Carlsbad, CA, USA). All other chemicals were purchased from Sigma‐Aldrich.

### 2.3 Cell culture

Human chondrosarcoma cell line (JJ012) was kindly provided by Dr. Sean P. Scully's laboratory (University of Miami School of Medicine, USA). Another human chondrosarcoma cell line (SW1353) was obtained from the American Type Culture Collection (Manassas, VA, USA). JJ012 and SW1353 cells were grown in Dulbecco's modified Eagle's medium (DMEM)/α‐MEM and DMEM supplemented with 20 mm HEPES, 10% heat‐inactivated fetal bovine serum, 2 mm glutamine, 100 U·mL^−1^ penicillin, and 100 μg·mL^−1^ streptomycin, respectively. All cells were maintained in a humidified incubator at 37 °C, 5% CO_2_.

### 2.4 Transwell assay

The cell migratory ability was determined using the transwell plates (Costar, NY, USA) as described previously with some modifications (Tsai *et al*., 2015). Briefly, cells were placed into the upper chamber and various concentrations of S1P were placed in the lower chamber. After incubation for 16 h at 37 °C in 5% CO_2_, cells were fixed in 3.7% formaldehyde solution and stained by 0.05% crystal violet, and the lower cells were then counted.

### 2.5 Small interfering RNA (siRNA) transfection

Cells were transfected with siRNA according to the manufacturers’ recommendations on standard procedure. Cells were transfected with siRNA using Lipofectamine 2000 reagent. The mRNA knockdown efficiency was confirmed by real‐time PCR or immunoblotting assay as described in the following sections.

### 2.6 Overexpression of SphK1

The pCMV plasmids harboring the human SphK1 open reading frame (ORF) were obtained from OriGene Technologies. The 293T cells were incubated with lentiviral supernatants prior to incubation with pCMV‐SphK1ORF plasmids for 24 h, and then incubated with fresh medium for another 48 h, followed by collecting their medium. The JJ012 cells were incubated with this conditional medium for 24 h. For stable cell lines, cells were selected by neomycin.

### 2.7 Reverse transcription (RT) and real‐time PCR

Total RNA was extracted using a TRIzol kit as described previously (Tsai *et al*., 2015). Briefly, the reverse transcription reaction was performed using the oligo (dT) primer. Real‐time PCR analysis was carried out using SYBR with sequence‐specific primers. The GAPDH mRNA expression was used as an internal control.

For miRNA detection, reverse transcription was performed using Mir‐X™ miRNA First‐Strand Synthesis and SYBR® RT‐PCR with the specific forward primer of miR‐101 (5′‐TACAGTACTGTGATAACTGAA‐3′). The U6 snRNA was used for normalization. The threshold was set above the nontemplate control background and within the linear phase of target gene amplification to calculate the cycle number at which the transcript was detected (denoted as CT).

### 2.8 Immunoblotting assay

Protein was isolated from human chondrosarcoma cells and its concentration was then determined as described previously (Huang *et al*., 2014; Tsai *et al*., 2015). Proteins were resolved by SDS/PAGE and transferred to the Immobilon polyvinylidene fluoride membranes. After incubation with primary and secondary antibodies, the membranes were visualized by enhanced chemiluminescence using Kodak X‐OMAT LS film.

### 2.9 Patients and specimen preparation

The study protocol was approved by the Institutional Review Board of China Medical University Hospital. All patients gave written consent before enrollment. The specimens of normal cartilage or tumor tissue were obtained from patients who were diagnosed with osteoarthritis or chondrosarcoma and underwent surgical resection at China Medical University Hospital. The histological grades (on a scale of I to III) of each chondrosarcoma patients were according to Jo and Doyle (2016).

### 2.10 Immunohistochemical (IHC) staining

The protein expression of SphK1 was determined on tissue slides using IHC staining as described previously (Tsai *et al*., 2015). Briefly, these tissue sections were deparaffinized with xylene and rehydrated through the addition of ethanol. Endogenous peroxidase activity was blocked with 3% hydrogen peroxide. After antigen retrieval by heating at 95 °C, the antibody against SphK1 was applied. The antibody‐binding signal was then detected using the NovoLink Polymer Detection System (Leica Microsystems, Heidelberg, Germany) and visualized using 3‐3′‐diaminobenzidine. The sections were counterstained with hematoxylin.

### 2.11 Plasmid construction and luciferase activity assay

The 3′ untranslated regions (3′UTRs) of the human TIMP‐3 gene were amplified by PCR using the following primer: 5′‐GGTTTAAACCACATCCCCTCTGTTAGG‐3′/5′‐GGCTCGAGCAGCCTACACATGACACAAGA‐3′. The 3′UTRs of TIMP‐3 were cloned downstream of the reporter gene in the pGL2‐Control vector. The predicted TIMP‐3 binding site for miRNA was identified by the Targetscan (http://www.targetscan.org/). Mutant plasmids that attenuate the interaction between TIMP‐3 3′UTR and miRNA were generated using a QuikChange Site‐Directed Mutagenesis kit (Stratagene, Cedar Creek, TX, USA). These plasmids were transfected into cells using Lipofectamine 2000. Following transfection, these cells were incubated with the indicated agents. Cell extracts were prepared and used for measuring the luciferase and β‐galactosidase activities.

### 2.12 *In vivo* tumor xenograft study

JJ012 cells that constitutively expressed pLenti CMV V5‐Luc were co‐transfected with pCMV plasmids alone or harboring human SphK1 ORF, JJ012/Luc, or JJ012/Luc‐SphK1, respectively. These cells (2 × 10^6^) that were resuspended in 50% of serum‐free DMEN/α‐MEM and 50% of Matrigel were intravenously injected into the lateral tail vein of severe combined immunodeficiency (SCID) mice. Lung metastasis was monitored using an *in vivo* imaging system (Xenogen IVIS imaging system). After six weeks, the mice were humanely sacrificed and the tumor tissues were removed and photographed. The protein and mRNA expressions of SphK1 were determined by IHC and real‐time PCR analyses, respectively.

### 2.13 Statistical analysis

All data are presented as mean ± standard error of the mean (SEM). Statistical analysis between the two samples was performed using the Student's *t*‐test. *P *<* *0.05 was considered significant.

## 3 Results

### 3.1 S1P inhibits cell migratory ability through the downregulation of MMP‐2 expression in human chondrosarcoma cells

**Figure 1:**
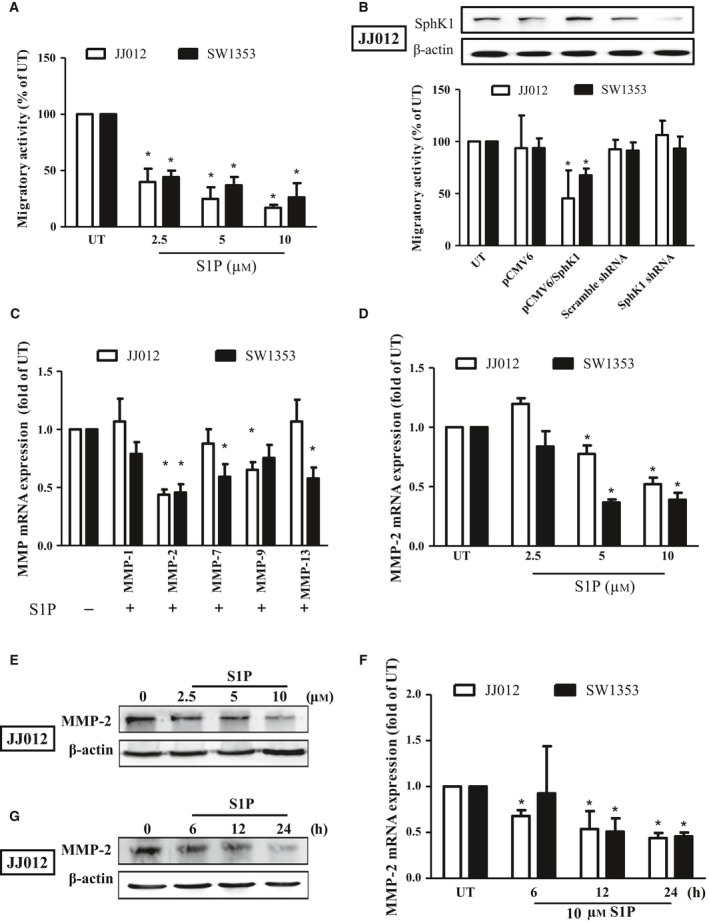
S1P inhibits migration through the downregulation of MMP‐2 in human chondrosarcoma cells. Starved JJ012 (open bar) and SW1353 (closed bar) cells were incubated with S1P (2.5–10 μm) for 24 h (A), or transfected with plasmids alone or plasmids containing SphK1 coding sequence or SphK1 shRNA (B), and the migratory ability was measured by the transwell assay. The overexpression and knockdown efficiencies of SphK1 were then determined by immunoblotting in JJ012 cells (B). (C) The JJ012 and SW1353 cells were incubated with 10 μm S1P for 24 h, and the mRNA expressions of MMP‐1, MMP‐2, MMP‐7, MMP‐9, and MMP‐13 were measured by real‐time PCR. JJ012 and SW1353 cells were treated with various concentrations of S1P for 24 h (D and E) or with 10 μm of S1P for different time periods (F and G); the MMP‐2 mRNA expressions were then determined by real‐time PCR. The MMP‐2 protein expressions following S1P stimulation were determined by immunoblotting in JJ012 cells (E and G). The results are expressed as mean ± SEM. *P < 0.05 compared with untreated control (*n* ≧ 3). UT, untreated control.

To investigate the migratory effect of S1P on human chondrosarcoma cells, S1P was applied to JJ012 and SW1353, the human chondrosarcoma cell lines. Results indicated that the cell migratory ability was found to be decreased significantly upon S1P stimulation via a concentration‐dependent manner in both JJ012 and SW1353 cells (Fig. 1A). It has been shown that S1P is generated from sphingosine inside the cell by SphK1. To study whether the endogenous S1P also affects cell migration, JJ012 and SW1353 cells were transfected with plasmids harboring SphK1 cDNA or SphK1 shRNA. Results showed that overexpression of SphK1 inhibited cell migration in human chondrosarcoma cells, while downregulation of SphK1 did not influence the cell migration (Fig. 1B). The sphingolipid metabolites, including S1P, sphingosine, and ceramide, are interconvertible (Bartke and Hannun, 2009). Moreover, most studies indicated that S1P showed the opposite role to sphingosine and ceramide, including cell survival, proliferation, and migration (Sharma and Prakash, 2017). To investigate whether the S1P‐inhibited chondrosarcoma cell migration resulted from attenuating cell viability, MTT analysis was applied. The results indicated that ceramide, but not S1P suppresses JJ012 and SW1353 cell viability (Fig. S1A,B). To determine whether the TIMP‐3 and MMP‐2 expressions were also regulated by the sphingolipid metabolites, sphingosine and ceramide were applied. The results revealed that only S1P upregulates TIMP‐3 and suppresses MMP‐2 expression in JJ012 and SW1353 cells (Fig. S1C,D). Interestingly, the TIMP‐3 and MMP‐2 expressions were both inhibited after ceramide stimulation, which may be resulted from attenuating cell viability by ceramide (Fig. S1B). To further confirm that the S1P‐regulated TIMP‐3 and MMP‐2 expressions were not due to interconversion other sphingolipid metabolites, a ceramide synthase inhibitor, fumonisin b1 (FB1), was used. Pretreatment with FB1 failed to suppress the S1P‐induced TIMP‐3 expression in JJ012 and SW1353 cells (Fig. S1E). Meanwhile, the S1P‐inhibited MMP‐2 expression was not rescued upon FB1 stimulation (Fig. S1F). Taken together, these results further confirm the direct effect of S1P on cell migration inhibition in human chondrosarcoma cells.

Previous studies have shown that the regulation of MMPs is responsible for the progression and metastatic ability of cancer, including chondrosarcoma (Deryugina and Quigley, 2015; Tsai *et al*., 2015). To investigate whether the regulation of MMPs was involved in the S1P‐inhibited chondrosarcoma migration, several MMP mRNA was monitored. Results showed that MMP‐2 mRNA and protein expression were found to be suppressed following S1P stimulation in both JJ012 and SW1353 cells (Fig. 1C) in the dose‐dependent (Fig. 1D,E) and time‐dependent manners (Fig. 1F,G). Moreover, MMP‐7 and MMP‐13 mRNA expressions were also found to be decreased in SW1353 cells and MMP‐9 mRNA expression was suppressed in JJ012 cells after S1P treatment (Fig. 1C). Taken together, these results indicated that S1P inhibits human chondrosarcoma cell migration through downregulating MMP‐2 expression.

### 3.2 S1P inhibits MMP‐2 expression through the upregulation of TIMP‐3 expression in human chondrosarcoma cells

**Figure 2:**
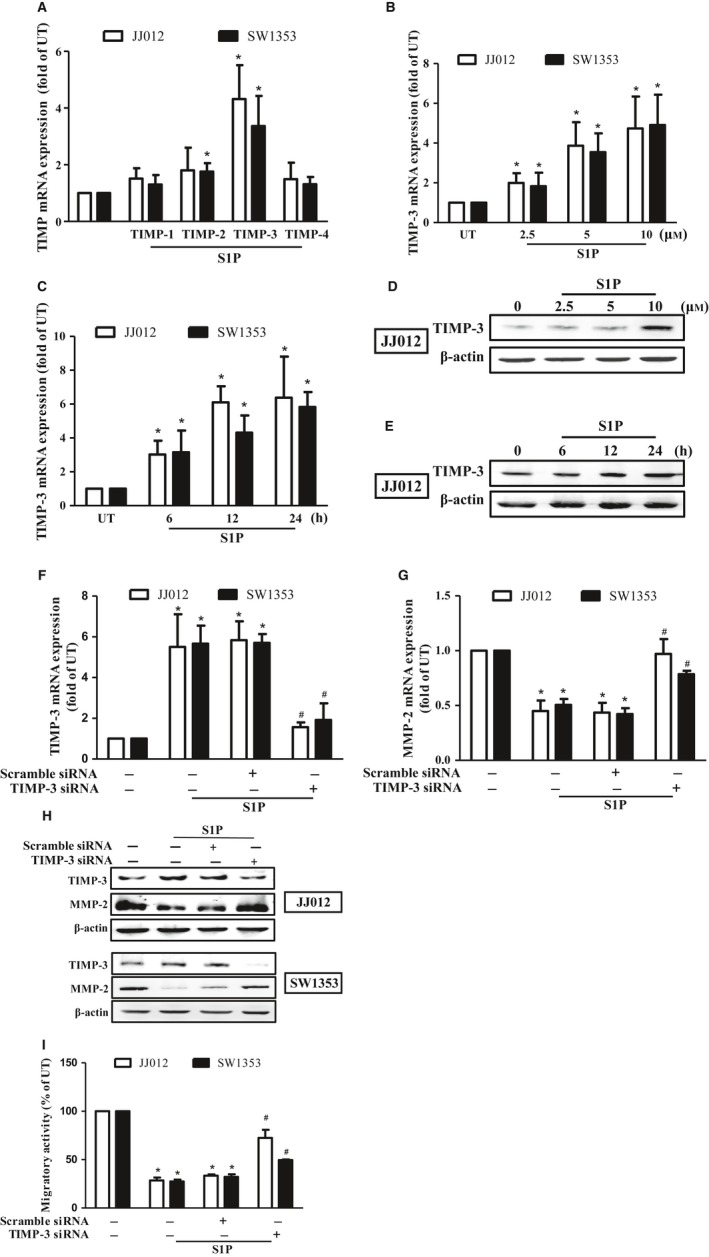
S1P inhibits migration through upregulating TIMP‐3 expression in human chondrosarcoma cells. (A) Starved JJ012 (open bar) and SW1353 (closed bar) cells were incubated with 10 μm of S1P, and the mRNA expressions of TIMP‐1–4 were determined by real‐time PCR. JJ012 and SW1353 cells were treated with various concentrations of S1P for 24 h (B and D) or with 10 μm of S1P for different time periods (C and E); the TIMP‐3 mRNA expressions were then determined by real‐time PCR. The TIMP‐3 protein expressions following S1P stimulation were determined by immunoblotting in JJ012 cells (D and E). The JJ012 and SW1353 cells were pretransfected with scramble siRNA or TIMP‐3 siRNA, and the cell migratory activity (I), TIMP‐3 mRNA (F), MMP‐2 mRNA (G), and their protein expressions (H) were determined by transwell, real‐time PCR, and immunoblotting analyses, respectively. The results are expressed as mean ± SEM. *P < 0.05 compared with untreated control; ^#^ *P* < 0.05 compared with the S1P‐treated group (*n* ≧ 3). UT, untreated control.

It has been shown that the activity of MMPs can be regulated by TIMPs (Baker *et al*., 2002). To investigate whether TIMPs were involved in the S1P‐inhibited MMP‐2 expression and the subsequent cell migration, the expressions of TIMP‐1–4 were determined. Results revealed that TIMP‐3 expression was found to be obviously increased (Fig. 2A) in the concentration‐ and time‐dependent manners upon S1P stimulation (Fig. 2B–E) in JJ012 and SW1353 cells. To further confirm whether the TIMP‐3 is involved in the S1P‐inhibited MMP‐2 expression and migration, TIMP‐3‐specific siRNA was applied. Results indicated that the knockdown of TIMP‐3 (Fig. 2F,H) obviously rescued the S1P‐inhibited MMP‐2 expression (Fig. 2G,H) and the subsequent migration (Fig. 2I). Taken together, these results revealed that S1P inhibits cell migration by downregulation of MMP‐2 expression through upregulating TIMP‐3 expression in human chondrosarcoma cells.

### 3.3 S1P induces TIMP‐3 expression through downregulation of microRNA (miRNA)‐101 expression in human chondrosarcoma cells

**Figure 3:**
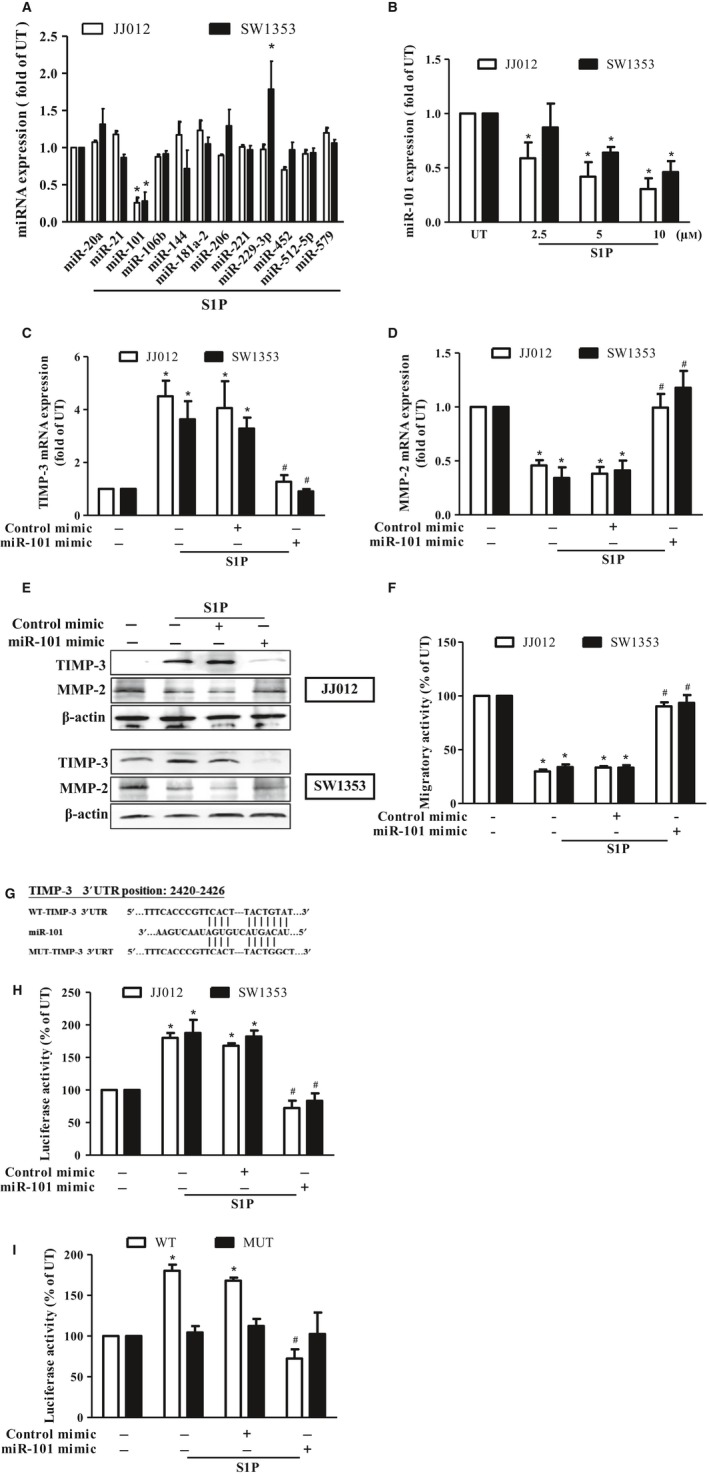
S1P‐induced TIMP‐3 expression is mediated through suppressing microRNA (miR)‐101 expressions in human chondrosarcoma cells. (A) Starved JJ012 (open bar) and SW1353 (closed bar) cells were incubated with 10 μm S1P for 24 h, and the expressions of miR‐20a, miR‐21, miR‐101, miR‐106b, miR‐144, miR‐181a‐2, miR‐206, miR‐221, miR‐229‐3p, miR‐452, miR‐512‐5p, and miR‐579 were measured by real‐time PCR. (B) JJ012 and SW1353 cells were treated with various concentrations (2.5–10 μm) of S1P for 24 h, and the expressions of miR‐101 were determined by real‐time PCR. The JJ012 and SW1353 cells were pretransfected with control or miR‐101 mimics for 24 h, followed by incubating with 10 μm of S1P for another 24 h, and the mRNA expressions of TIMP‐3 (C) and MMP‐2 (D) as well as their protein expressions (E) and the migratory activity (F) were then determined by real‐time PCR, immunoblotting, and transwell analyses, respectively. (G) The luciferase reporter vectors harboring the WT 3′ UTR (WT‐TIMP‐3 3′ UTR) and nucleotides mutated in this region (MUT‐TIMP‐3 3′ UTR) of TIMP‐3 were constructed. (H) JJ012 and SW1353 cells were co‐transfected with the luciferase plasmid harboring WT‐TIMP‐3 3′ UTR and miR‐101 or control mimics for 24 h, followed by treatment with 10 μm of S1P for another 24 h, and the relative luciferase activity was then determined. (I) JJ012 cells were co‐transfected with the luciferase plasmids harboring either WT or MUT‐TIMP‐3 3′ UTR and the mimics of control or miR‐101 for 24 h, followed by treatment with 10 μm of S1P for another 24 h, and the relative luciferase activity was then determined. Cells without treatment were used as the untreated control (set to 1 or 100), and data were shown as multiples of that. The results are expressed as mean ± SEM. *P <* *0.05 compared with UT; ^#^ *P *<* *0.05 compared with the S1P‐treated group (*n* ≧ 3). UT, untreated control; 3′ UTR, 3′ untranslated region; WT, wild‐type; MUT, mutant.

MicroRNA (miRNA) was shown to play important roles in the regulation of cancer progression and metastasis (Chen *et al*., 2015a; Hu *et al*., 2016; Nugent, 2015; Tsai *et al*., 2015). To investigate whether miRNA is responsible for the S1P‐induced TIMP‐3 expression, 12 predicted miRNA that contain binding sites of the 3′UTR of TIMP‐3, identified by Targetscan (http://www.targetscan.org/), were studied. Results showed that miR‐101 expression was significantly downregulated by S1P stimulation (Fig. 3A) in a dose‐dependent manner (Fig. 3B). To investigate the effects of miR‐101 on the S1P‐regulated TIMP‐3 expression and cell migration in human chondrosarcoma cells, the miR‐101 mimic was applied. Results indicated that the S1P‐induced TIMP‐3 mRNA and protein expressions were found to be inhibited upon miR‐101 mimic transfection (Fig. 3C,E), while transfection of miR‐101 mimic rescued the S1P‐inhibited MMP‐2 mRNA expression (Fig. 3D,E) and the subsequent migration (Fig. 3F) in the JJ012 and SW1353 cells. To determine whether miR‐101 directly targets the 3′UTR of TIMP‐3, the luciferase reporter vectors harboring wild‐type (WT) or mismatch mutant (MUT) of the 3′UTR of TIMP‐3 were constructed (Fig. 3G). These vectors were transfected into JJ012 and SW1353 cells followed by treatment with S1P and the luciferase activity was then assessed. The luciferase activity of human chondrosarcoma cells was found to be increased only in WT‐TIMP‐3 3′UTR transfection, but not in MUT‐TIMP‐3 3′UTR (Fig. 3I). Meanwhile, the S1P‐augmented TIMP‐3 luciferase activity was attenuated upon miR‐101 mimic transfection (Fig. 3H), indicating that the S1P‐induced TIMP‐3 expression is mediated by downregulating miR‐101 expression.

### 3.4 S1P induces TIMP‐3 expression through the c‐Src/MEK/ERK signaling axis in human chondrosarcoma cells

**Figure 4:**
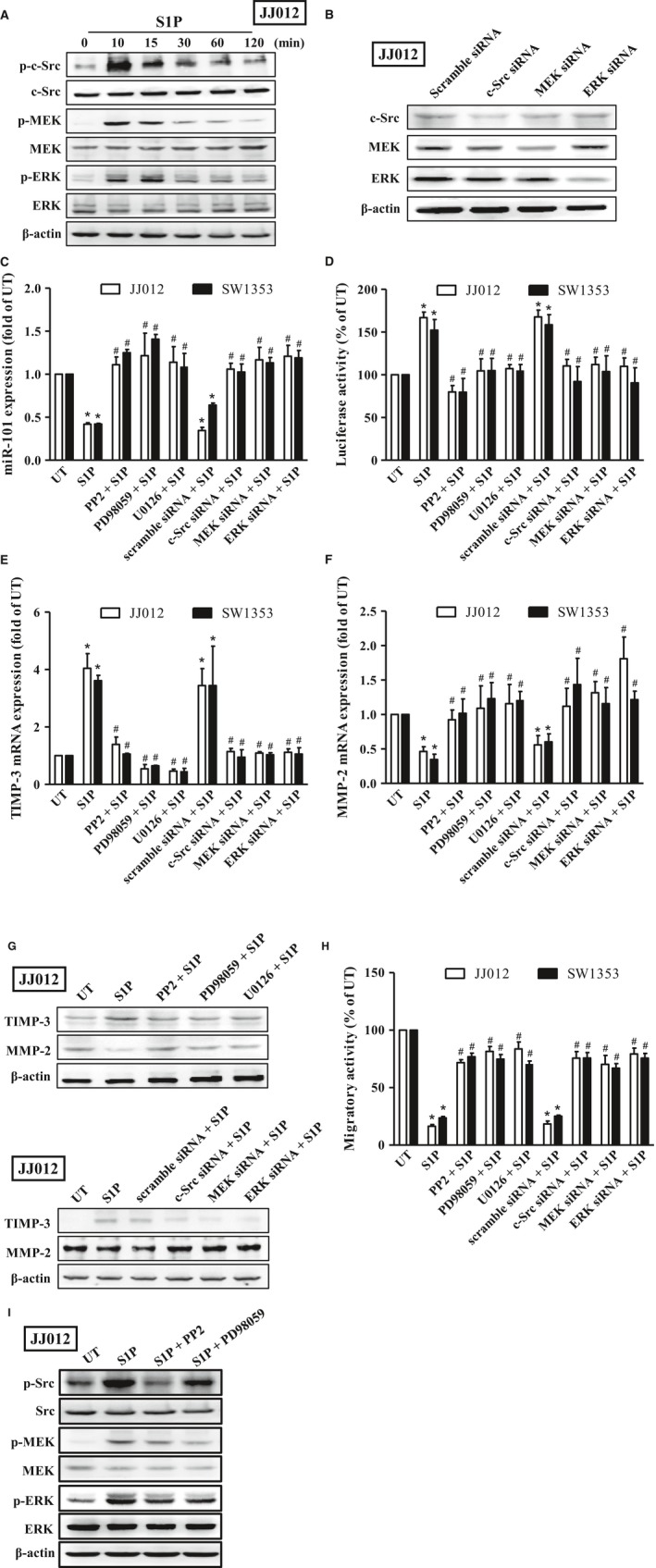
S1P‐induced TIMP‐3 expression is mediated through the c‐Src/MAPK signaling axis in human chondrosarcoma cells. (A) Starved JJ012 cells were incubated with 10 μm of S1P for 10, 15, 30, 60, and 120 min, and the phosphorylation of c‐Src, MEK, and ERK was examined by immunoblotting using anti‐phospho c‐Src, MEK, or ERK antibodies, followed by reprobing against total c‐Src, MEK, ERK, and β‐actin to show the equal loading amounts. (B) The JJ012 cells were transfected with siRNA of scramble, c‐Src, MEK, and ERK for 24 h, and the protein expressions of c‐Src, MEK, and ERK were then determined to show the knockdown efficiency. Starved JJ012 (open bar) and SW1353 (closed bar) cells were pretreated with 10 μm of PP2, PD98059, and U0126 for 1 h or transfected with siRNA of scramble, c‐Src, MEK, and ERK for 24 h, followed by treatment with 10 μm of S1P for another 24 h, and expressions of miR‐101 (C), TIMP‐3 (E), and MMP‐2 (F) mRNA, as well as the protein expressions of TIMP‐3 and MMP‐2 (G), and migratory activity (H), were then determined by real‐time PCR, immunoblotting, and transwell analyses, respectively. (D) JJ012 and SW1353 cells were transfected with plasmids harboring with WT‐TIMP‐3 3′UTR, followed by treatment with 10 μm of PP2, PD98059, and U0126 for 1 h or transfected with siRNA of scramble, c‐Src, MEK, and ERK for 24 h prior to incubation with 10 μm of S1P for another 24 h. The promoter activity of TIMP‐3 was then determined by luciferase activity assay. (I) Starved JJ012 cells were pretreated with 10 μm of PP2 and PD98059 for 1 h, followed by treatment with 10 μm of S1P for another 10 min, and the phosphorylation of Src, MEK, and ERK was then determined by immunoblotting analysis. Cells without treatment were used as the untreated control (set to 1 or 100), and data were shown as multiples of that. The results are expressed as mean ± SEM. *P <* *0.05 compared with UT; ^#^ *P *<* *0.05 compared with the S1P‐treated group (*n* ≧ 3). UT, untreated control; 3′ UTR, 3′ untranslated region; WT, wild‐type.

Previous studies have revealed that Src signaling is responsible for the S1P‐induced cell migration in MCF‐7, HUVECs, and Capan‐1 cells (Guo *et al*., 2015; Huang *et al*., 2009; Lucki and Sewer, 2011). Additionally, the MEK/ERK signaling axis contributes to the Src‐dependent tumor growth and migration (Wu *et al*., 2012; Zhang *et al*., 2012). To investigate whether Src signaling is involved in the S1P‐regulated TIMP‐3 expression and the subsequent cell migration in human chondrosarcoma cells, the phosphorylations of c‐Src, MEK, and ERK were monitored. Results showed that c‐Src, MEK, and ERK were phosphorylated within 10 min poststimulation of S1P and declined with time in JJ012 cells (Fig. 4A). Moreover, the S1P‐inhibited miR101 expression was rescued upon the inhibition of Src, MEK, or ERK by their specific siRNA (Fig. 4B) or inhibitors in JJ012 and SW1353 cells (Fig. 4C). Meanwhile, the S1P‐induced TIMP‐3 expression was inhibited following blockage of Src, MEK, or ERK, as demonstrated by promoter activity assay (Fig. 4D), real‐time PCR (Fig. 4E), and immunoblotting (Fig. 4G). Subsequently, the S1P‐suppressed chondrosarcoma cell migratory ability and MMP‐2 expression were reversed when Src, MEK, and ERK are inhibited (Fig. 4F–H). Moreover, the effects of S1P on human chondrosarcoma were not regulated by either p38‐ or JNK‐dependent signaling pathway (Fig. S2). On the other hand, we also showed that the S1P‐induced MEK and ERK phosphorylations were inhibited by PP2, Src inhibitor. However, the inhibition of MEK signaling by PD98059 has no effects on S1P‐induced Src phosphorylation (Fig. 4I), indicating that the S1P‐induced MEK and ERK phosphorylations are Src dependent. Taken together, S1P‐inhibited cell migratory ability by upregulation of TIMP‐3 is performed through inducing the c‐Src/MEK/ERK signaling axis and then suppressing miR‐101 expression in human chondrosarcoma cells.

### 3.5 Overexpression of SphK1 decreases chondrosarcoma cell migration and metastasis *in vivo*

**Figure 5:**
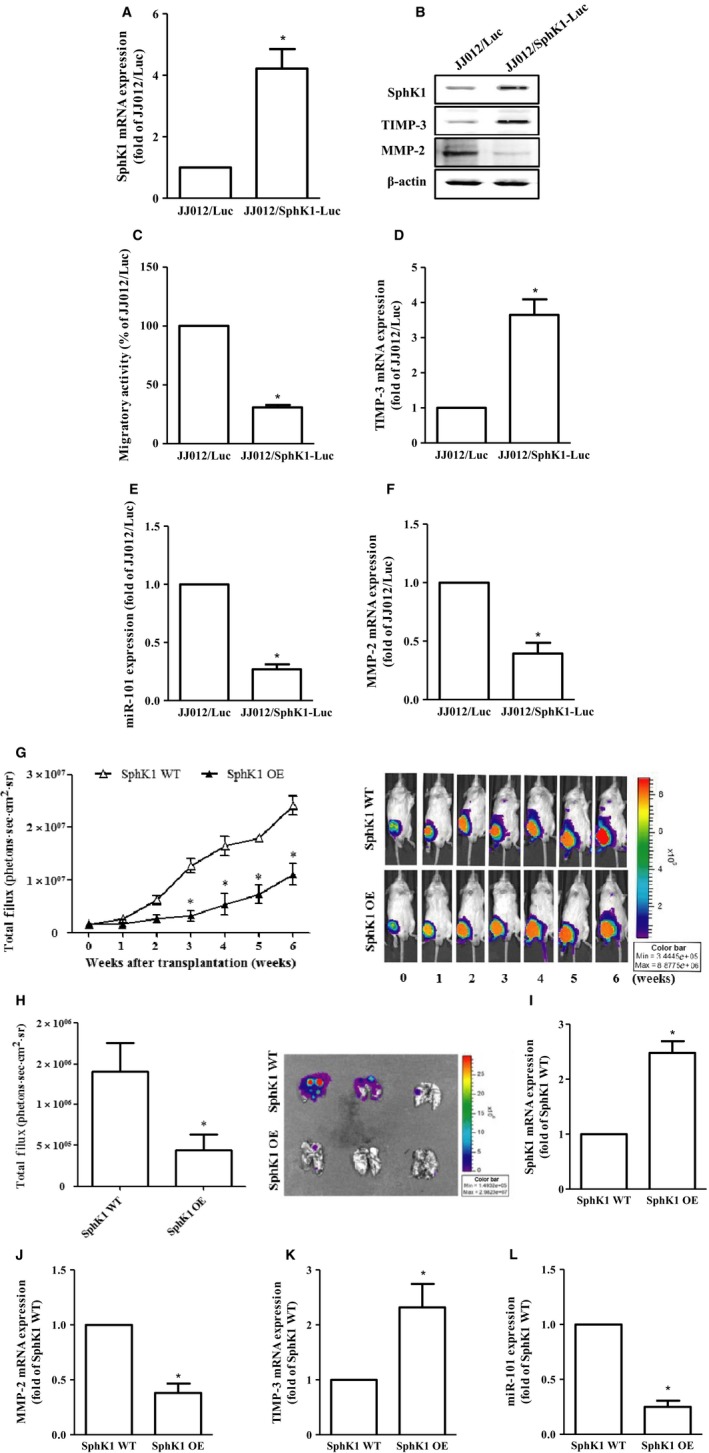
Overexpression of SphK1 decreases cell migration and chondrosarcoma metastasis *in vitro* and *in vivo*. The constitutively expressed pLenti CMV V5‐Luc JJ012 cells were transfected with pCMV6 plasmid alone (JJ012/Luc) or harboring with human SphK1 ORF cDNA (JJ012/Sphk1‐Luc), followed by the determination of the protein (B) and mRNA expressions of SphkK1 (A), TIMP‐3 (D), MMP‐2 (F), as well as miR‐101 expression (E) and the migratory activity (C) by immunoblotting, real‐time PCR, and the transwell analyses, respectively. (G) JJ012/Luc or JJ012/SphK1‐Luc cells representing the wild‐type or overexpression of SphK1 were injected into the lateral tail vein of severe combined immunodeficient mice, and the development of lung metastasis was monitored by bioluminescence imaging at the time intervals. These images were then quantified (photons/s of lung region). After six weeks, these mice were humanely sacrificed, and the lung tissues were excised, photographed, and quantified (H). The mRNA expressions of SphK1 (I), MMP‐2 (J), and TIMP‐3 (K), as well as miR‐101 (L) on these tumors, were then assessed by real‐time PCR analysis. Cells without overexpression of SphK1 were used as the control (set to 1 or 100), and data were shown as multiples of that. The results are expressed as mean ± SEM. *P <* *0.05 compared with control (*n* ≧ 3). SphK1, sphingosine kinase 1; ORF, open reading frame; WT, wild‐type; OE, overexpression.

To confirm the role of S1P in chondrosarcoma cell metastasis *in vivo*, we took advantage of JJ012 cells that stably express pLenti CMV V5‐Luc (JJ012/Luc) and transfected with SphK1 cDNA. Results showed that the protein and mRNA expressions of SphK1 were significantly overexpressed in JJ012 cells stably expressing SphK1 cDNA (JJ012/SphK1‐Luc) (Fig. 5A,B). In addition, the migratory ability (Fig. 5C), MMP‐2 expression (Fig. 5B,F), and miR‐101 expression (Fig. 5E) of JJ012/SphK1‐Luc cells were found to be significantly decreased compared to those in control cells (JJ012/Luc). However, the TIMP‐3 expression in JJ012/SphK1‐Luc cells was higher than that in JJ012/Luc cells (Fig. 5B,D). Moreover, the cell viability between JJ012/Luc and JJ012/SphK1‐Luc cells was almost the same (data not shown), indicating that overexpression of SphK1 cannot influence the cell viability. To examine the effect of endogenous S1P on metastasis of chondrosarcoma *in vivo*, JJ012/Luc or JJ012/SphK1‐Luc cells were intravenously injected into SCID mice through the lateral tail vein, and the tumor metastasis was monitored by bioluminescence imaging. Our results showed that overexpression of SphK1 significantly suppressed lung metastasis of chondrosarcoma *in vivo* (Fig. 5G). Mice were then humanely sacrificed after 6‐week injection; *ex vivo* imaging of lungs removed from the mice showed a higher luciferase intensity in the JJ012/Luc compared to that in the JJ012/SphK1‐Luc group (Fig. 5H). Moreover, the SphK1 expression is positively associated with TIMP‐3 expression *in vivo*, whereas the expressions of MMP‐2 and miR‐101 were found to be decreased in JJ012/SphK1‐Luc groups (Fig. 5I–L). Taken together, these results indicated that endogenous S1P suppresses miR‐101 expression, followed by augmenting TIMP‐3 induction and then preventing the MMP‐2‐dependent lung metastasis of chondrosarcoma *in vivo*.

### 3.6 SphK1 expression correlates with TIMP‐3 and miR‐101 in clinical specimen

**Figure 6:**
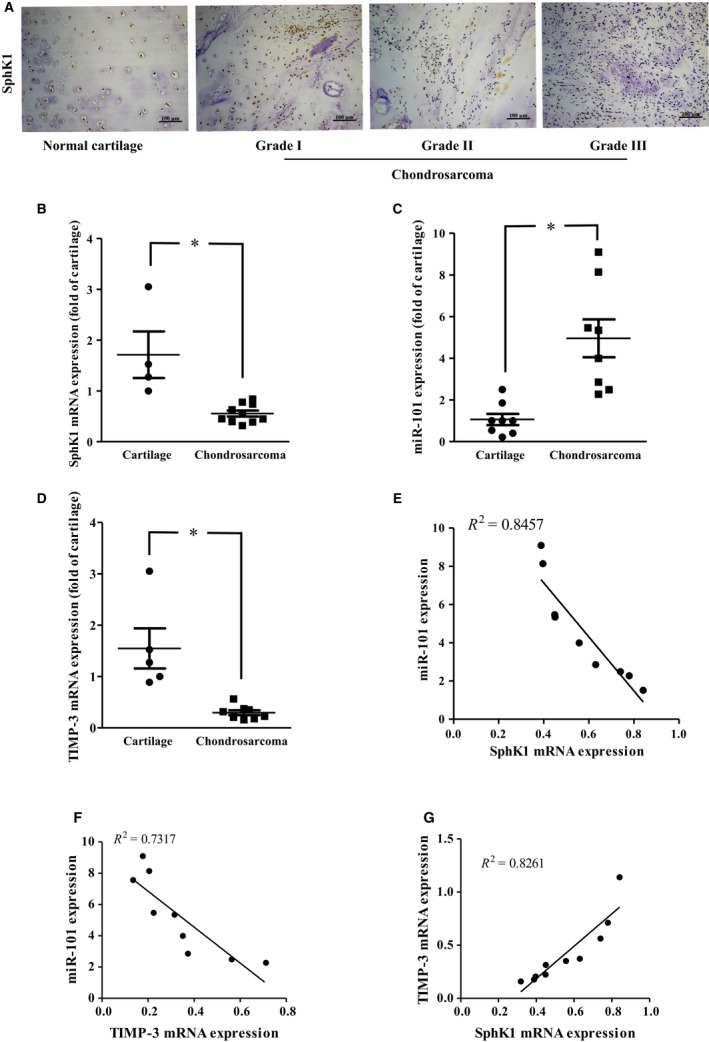
Clinical importance of SphK1, TIMP‐3, and miR‐101 in chondrosarcoma. (A) The protein expression of SphK1 was determined in the tissue array by IHC staining. The mRNA expressions of SphK1 (B) and TIMP‐3 (D), as well as miR‐101 (C) in normal cartilage and chondrosarcoma tissues, were examined by real‐time PCR. The correlation between miR‐101 and SphK1 (E), miR‐101 and TIMP‐3 (F), and TIMP‐3 and SphK1 (G) gene expressions was then determined. Quantitative results are expressed as the mean ± SEM. *P <* *0.05 compared with cartilage tissue samples (*n* ≧ 3).

To identify the correlation between SphK1, miR‐101, and TIMP‐3 expressions in clinical patients with chondrosarcoma, the biopsy samples of normal cartilage and chondrosarcoma patients were collected. The expression patterns of SphK1 on normal cartilage and chondrosarcoma patients were first assessed. The expressions of SphK1 in patients with chondrosarcoma were lower than those in normal individuals. Additionally, the expressions of SphK1 were negatively correlated with chondrosarcoma progression (Fig. 6A), indicating that SphK1 may be associated with clinicopathologic stages in chondrosarcoma. Meanwhile, the mRNA expressions of SphK1 and TIMP‐3 of chondrosarcoma tissue sample were lower than those of normal cartilage; however, the miR‐101 expression was opposite (Fig. 6B–D). Furthermore, we also demonstrated that the SphK1 and TIMP‐3 expressions were negatively correlated with miR‐101 (Fig. 6E,F). Meanwhile, the TIMP‐3 expression was positively correlated with TIMP‐3 in chondrosarcoma tissues (Fig. 6G). In summary, higher expressions of SphK1 and TIMP‐3 as well as lower expressions of miR‐101 and MMP‐2 were linked with chondrosarcoma development and metastasis.

## 4 Discussion

While the incidence is relatively rare, chondrosarcoma is still the second most common primary malignancy of bone cancer (Gelderblom *et al*., 2008). It is difficult to diagnose and insensitive to chemo‐ or radiotherapies. Therefore, the surgical excision of tumor is the only option for the therapy to chondrosarcoma (Leddy and Holmes, 2014). However, the high potential for local invasion and distant metastasis of chondrosarcoma is the challenge for this treatment. Thus, understanding or regulation of the metastatic processes on chondrosarcoma is a strategy for the treatment of this disease. Increasing evidence indicates that upregulation of MMPs is required for metastasis in several cancers, including chondrosarcoma, and is also responsible for their poor prognosis (Gweon and Kim, 2014). MMPs therefore represent the potential target for preventing cancer metastasis, especially targeting for MMP‐2. Herein, we showed that S1P inhibits MMP‐2 expression through the upregulation of TIMP‐3, thereby suppressing human chondrosarcoma cell migration. We also illustrated that S1P upregulates TIMP‐3 expression through the inhibition of miR‐101 via the c‐Src/MEK/ERK signaling axis in human chondrosarcoma cells. Furthermore, overexpression of SphK1 that generates intracellular S1P suppressed chondrosarcoma metastasis to lungs *in vivo*. Meanwhile, the expression levels of SphK1 were negatively correlated with miR‐101 and tumor grades of human chondrosarcoma. These results suggested that S1P and miR‐101 may be the novel molecular targets for inhibiting MMP‐2‐induced metastasis in chondrosarcoma.

S1P, a simple bioactive sphingolipid, is produced through the phosphorylation of sphingosine by SphKs. It regulates a variety of cancer‐related processes, including autophagy, proliferation, angiogenesis, and migration by binding to its membranous receptors or targeting to the intracellular molecules (Chang *et al*., 2009; Huang *et al*., 2009, 2011, 2014). S1P is also considered as a cancer‐promoting molecule, as the upregulation of SphK1 is a common phenomenon in several cancers and correlates with poor prognosis (Xu *et al*., 2016). Interestingly, we showed that the expression of SphK1 decreases with tumor grades. Moreover, S1P and SphK1 play negative roles on chondrosarcoma metastasis. Indeed, some studies also showed that S1P inhibits migration through its receptor S1P_2_ and the ROCK‐mediated vimentin S71 phosphorylation in MDA‐MB‐435D and C643 cells (Hyder *et al*., 2015). Also, S1P inhibits HUVEC angiogenesis through the binding to S1P_2_ and activates the downstream TIMP‐2 in coronary artery smooth muscle cells (Mascall *et al*., 2012). Furthermore, through the inactivation of Akt, S1P suppresses cell proliferation in keratinocytes (Kim *et al*., 2004) and human prostate cancer cells (Chang *et al*., 2009; Huang *et al*., 2014). These results suggest that whether S1P has a stimulatory or inhibitory effect on cells may be dependent on receptor composition or its intracellular downstream targets. Thus, which membranous receptors or intracellular targets are responsible for the S1P‐inhibited human chondrosarcoma metastasis needs further investigation.

Src, a proto‐oncogene encoding the nonreceptor tyrosine kinase, regulates several intracellular signaling pathways implicated in multistep processes of tumorigenesis, including the promotion of epithelial–mesenchymal transition, angiogenesis, migration, and invasion, as well as the inhibition of apoptosis (Chen *et al*., 2015b). At least three downstream signaling pathways are involved in the Src‐regulated cellular effects, including the mitogen‐activated protein kinase (MAPK), the phosphatidylinositol 3‐kinase (PI3K), and the signal transducer and activator of transcription 3 (STAT3) pathways (Wu *et al*., 2012; Zhang *et al*., 2012). The MAPK, including c‐Jun NH2‐terminal kinase (JNK), p38 MAPK, and extracellular signal‐regulated kinase (ERK), regulates various cancer‐related cellular activities, including promoting survival, proliferation, angiogenesis, as well as suppressing apoptosis (Kim and Choi, 2015). Interestingly, our results showed that the signaling axis of c‐Src/MEK/ERK induced by S1P is responsible for the inhibition of metastasis in human chondrosarcoma cells. These results are similar to previous studies showing that the activation of ERK is involved in the inhibition of angiogenesis (Chetty *et al*., 2008) and activation of apoptosis (Yang *et al*., 2016) in tumor cells. On the other hand, we showed that the ERK‐mediated metastasis‐inhibiting effects resulted from inducing TIMP‐3 expression in human chondrosarcoma cells. Previous studies showed similar results on TIMP‐3 regulation which is induced by ERK phosphorylation and the downstream transcriptional factor, Sp1 in human lung cancer cells (Chetty *et al*., 2008).

Accumulating evidence revealed that miRNA regulate several cancer‐related processes during tumorigenesis through binding to the 3′UTR of the target genes (Nugent, 2015; Tsai *et al*., 2015). Furthermore, miR‐101 was considered to regulate cell proliferation, angiogenesis, invasion, and migration in many types of tumors and serve as a tumor suppressor (Li *et al*., 2015; Liu *et al*., 2014; Ma *et al*., 2016). In this study, we showed that extracellularly applied S1P or overexpressing SphK1 inhibit miR‐101 expression in human chondrosarcoma cells. The other study demonstrated that miR‐101 downregulates Sphk1 expression in colorectal cells (Chen *et al*., 2015a). Thus, the expression of miR‐101 and Sphk1 was negatively regulated. Indeed, we showed that the Sphk1 decreased in chondrosarcoma tissue, while miR‐101 increased. These observations suggested that the Sphk1 and miR‐101 are the markers for chondrosarcoma progression. Thus, S1P may develop as a novel potential target and provide a new miRNA‐based molecular diagnosis for human chondrosarcoma treatment.

The hydrolysis of the ECM appears to facilitate tumor cell migration contributing to the metastatic dissemination of malignant cells (Deryugina and Quigley, 2015). The major group of proteases that participate in ECM remodeling are MMPs. Hence, the expression and activities of MMPs are tightly controlled in normal circumstances (Ra and Parks, 2007). Abnormal activation or overexpression of MMPs are noted in many pathological processes involved in tumorigenesis (Ra and Parks, 2007; Tsai *et al*., 2015). Therefore, MMPs are considered as the therapeutic targets for cancer therapy. The activities of MMPs can be regulated intracellularly by their specific inhibitors, such as TIMPs, which bind the catalytic domain of MMPs in a 1 : 1 stoichiometric interaction (Baker *et al*., 2002; Ra and Parks, 2007). TIMP‐3 is sequestered to ECMs, whereas all other TIMPs (TIMP‐1, 2, and 4) are present in the soluble forms (Baker *et al*., 2002). Previous studies have been shown that TIMP‐3 regulates the activities of MMPs, such as MMP‐2, ‐7,‐9, and ‐13 (Deb *et al*., 2015; Fu *et al*., 2016; Hu *et al*., 2016; Yamamoto *et al*., 2016). Herein, we also showed that S1P inhibits cell migration mainly by downregulation of MMP‐2, but partially by MMP‐7, ‐9, and ‐13. It may have resulted from the induction of TIMP‐3 expression in human chondrosarcoma cells. This issue needs to be further investigated.

## 5 Conclusions

Taken together, although S1P has been considered as a cancer‐promoting molecule in several studies, others showed the negative roles on tumorigenesis. Herein, we provide the evidence that S1P inhibits chondrosarcoma metastasis *in vitro* and *in vivo*. Additionally, the S1P‐inhibited metastasis resulted from upregulating TIMP‐3 expression through suppressing miR‐101 via the c‐Src/MEK/ERK signaling pathway. These results suggested that S1P may represent a promising new target for treating chondrosarcoma.

## Author contributions

YL Huang and CH Tang participated in the conception and design of the experiments. CH Tsai, DY Yang, CY Lin, and YL Huang performed the experiments. CH Tsai provided human samples analyzed in the study. TM Chen and CH Tang developed methodology. DY Yang, CY Lin, and YL Huang were involved in data acquisition. CH Tang and YL Huang analyzed and interpreted data. YL Huang wrote, reviewed, and/or revised the manuscript.

## Supporting information


**Fig. S1.** The TIMP‐3 and MMP‐2 expression were not regulated by other sphingolipid metabolites in human chondrosarcoma cells.
**Fig. S2.** The S1P‐inhibited human chondrosarcoma cell migration is not mediated through p38‐ and JNK‐dependent pathway.
**Fig. S3.** The cell migration, TIMP‐3 mRNA, MMP‐2 mRNA, and miR‐101 expression were not regulated by chemical inhibitor or their siRNA stimulation in JJ012 and SW1353 cells.

**Doc. S1.** Supplementary results.
