# Supplementary figures and images for: Sphingosine‐1‐phosphate suppresses chondrosarcoma metastasis by upregulation of tissue inhibitor of metalloproteinase 3 through suppressing miR‐101 expression
Source: Mol Oncol. 2017 Aug 8;11(10):1380–98. doi: 10.1002/1878-0261.12106 (PMC5623823; doi:10.1002/1878-0261.12106)

Supplementary Figure 1

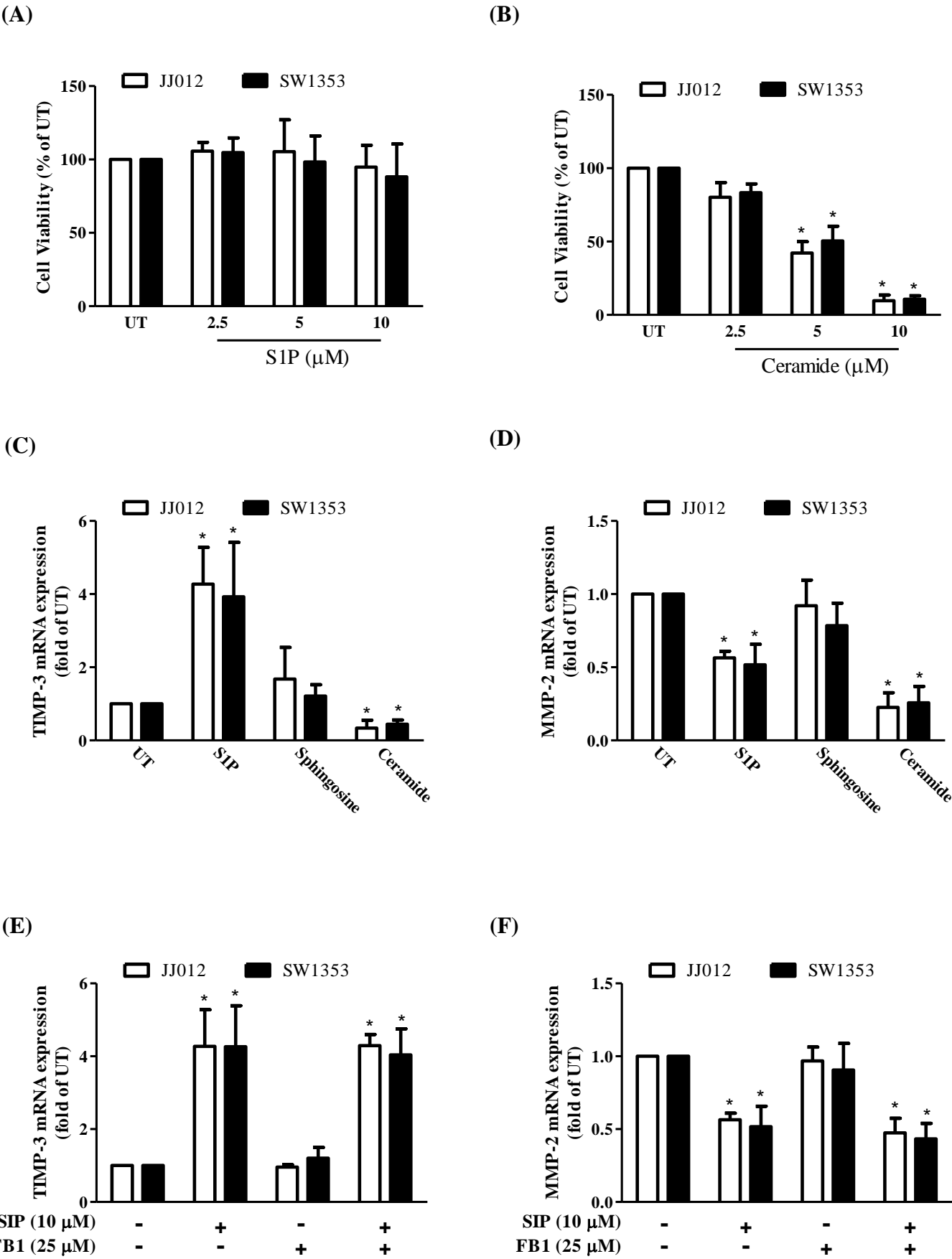

Supplementary Figure 2

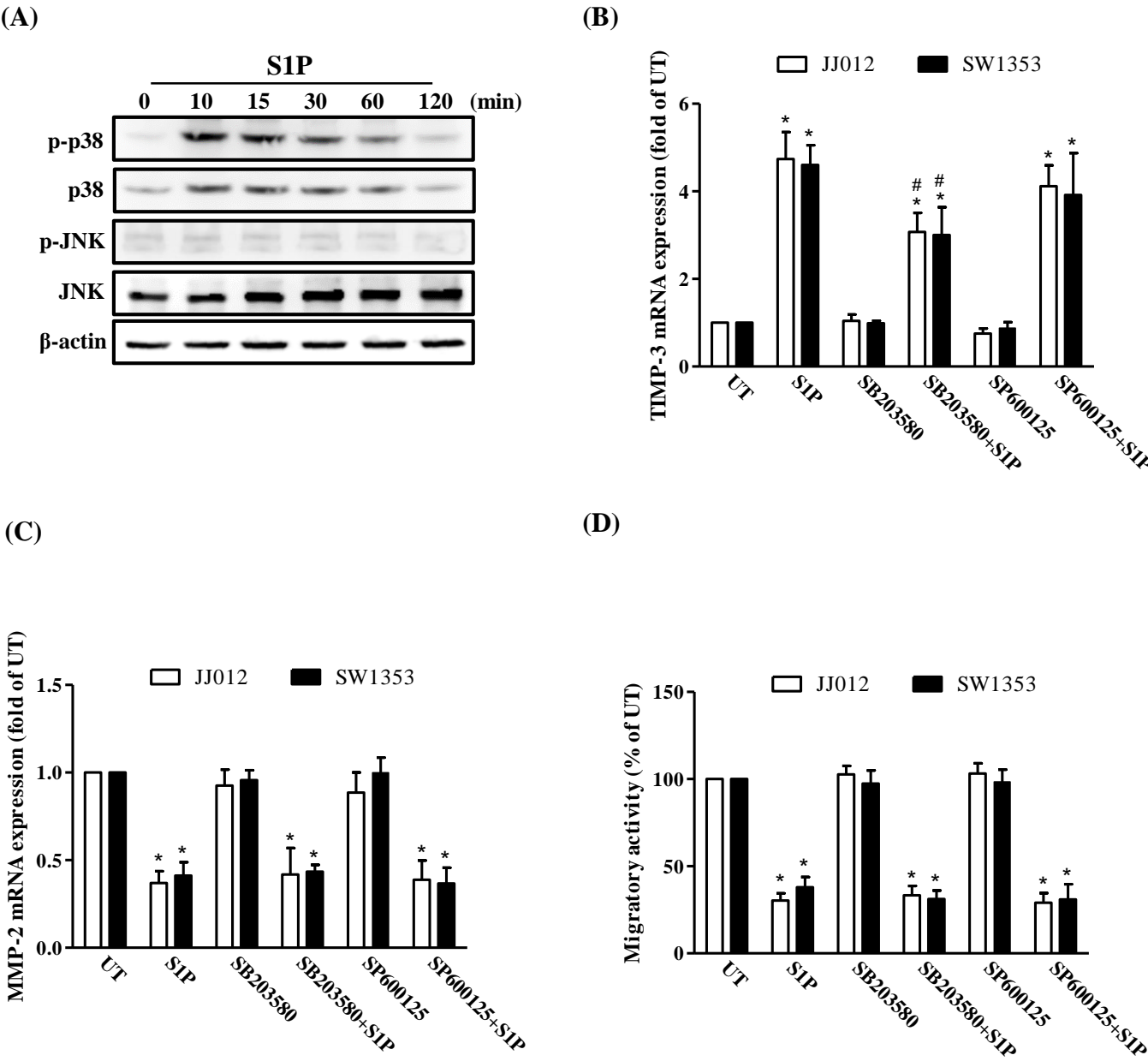

Supplementary Figure 3

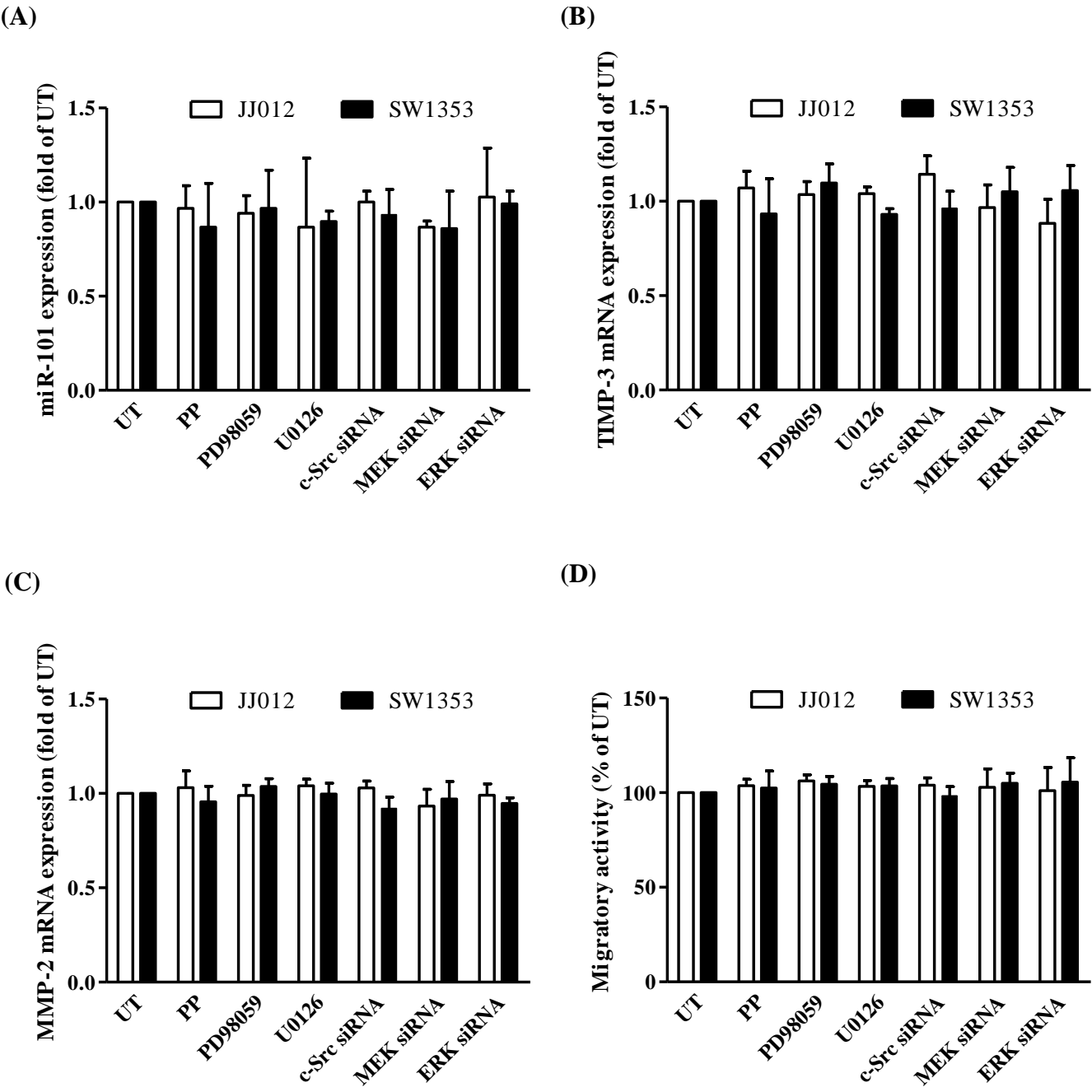

Supplement: Supplementary file 1 — Fig. S1. The TIMP‐3 and MMP‐2 expression were not regulated by other sphingolipid metabolites in human chondrosarcoma cells. Fig. S2. The S1P‐inhibited human chondrosarcoma cell migration is not mediated through p38‐ and JNK‐dependent pathway. Fig. S3. The cell migration, TIMP‐3 mRNA, MMP‐2 mRNA, and miR‐101 expression were not regulated by chemical inhibitor or their siRNA stimulation in JJ012 and SW1353 cells. [file MOL2-11-1380-s001.pdf]
