# Supplementary material for: Sphingosine‐1‐phosphate suppresses chondrosarcoma metastasis by upregulation of tissue inhibitor of metalloproteinase 3 through suppressing miR‐101 expression
Source: Mol Oncol. 2017 Aug 8;11(10):1380–98. doi: 10.1002/1878-0261.12106 (PMC5623823; doi:10.1002/1878-0261.12106)
Supplement: Supplementary file 2 — Doc. S1. Supplementary results. [file MOL2-11-1380-s002.docx]

**Supplementary Results**

*The sphingolipid metabolites can’t attenuate TIMP-3 and MMP-2 expression in chondrosarcoma cells*

Since the sphingolipid metabolites, including S1P, sphingosine, and ceramide, are interconvertible ([Bartke and Hannun, 2009](#_ENREF_1)). Moreover, most studies indicated that S1P showed the opposite role than sphingosine and ceramide, including cell survival, proliferation, and migration, which is called sphingolipid rheostat ([Sharma and Prakash, 2017](#_ENREF_2)). To investigate whether the S1P-inhibited chondrosarcoma cell migration is resulted from attenuating cell viability, MTT analysis was applied. The results indicated that ceramide (Supplementary Figure 1B), but not S1P (Supplementary Figure 1A) suppresses JJ012 and SW1353 cell viability. To determine whether the TIMP-3 and MMP-2 expressions were also regulated by the sphingolipid metabolites, sphingosine and ceramide were applied. The results showed revealed that only S1P upregulates TIMP-3 and suppresses MMP-2 expression in JJ012 and SW1353 cells (Supplementary Figure 1C and 1D). Interestingly, the TIMP-3 and MMP-2 expressions were both inhibited after ceramide stimulation, which may be resulted from attenuating cell viability by ceramide (Supplementary Figure 1B). To further confirm the S1P-regulated TIMP-3 and MMP-2 expressions were not resulted from interconverting to other sphingolipid metabolites, a ceramide synthase inhibitor, fumonisin b1 (FB1), was used. Pretreatment with FB1 failed to suppress the S1P-induced TIMP-3 expression in JJ012 and SW1353 cells (Supplementary Figure 1E). Meanwhile, the S1P-inhibited MMP-2 expression was not rescued upon FB1 stimulation (Supplementary Figure 1F). Taken together, these results further confirm the direct effect of S1P on cell migration inhibition in human chondrosarcoma cells.

*The p38 and JNK signaling were not responsible for the S1P-inhibited cell migration in human chondrosarcoma cells*

To investigate whether the MAPK signaling pathway was involved in the S1P-attenulated cell migration in human chondrosarcoma cells, the p38 and JNK pathways were monitored. Like ERK phosphorylation showed in figure 4A, p38, but not JNK, was phosphorylated following S1P stimulation (Supplementary Figure 2A). To further confirm the p38 and JNK pathways were participated in the S1P-inhibited human chondrosarcoma cell migration, their specific inhibitors were applied. Results showed that either pretreatment with SB203580 or SP600125, the p38 and JNK specific inhibitors, respectively, failed to rescue the S1P-inhibited MMP-2 expression and cell migration (Supplementary Figure 2C and 2D). Moreover, inhibition of JNK also failed to reverse the S1P-induced TIMP-3 expression in JJ012 and SW1353 cells (Supplementary Figure 2B). Interestingly, inhibition of p38 partially suppressed the S1P-induced TIMP-3 expression, but still have TIMP-3 induction (Supplementary Figure 2B), suggesting that the S1P-induced TIMP-3 is partially mediated through p38 signaling pathway, but it is not responsible for the subsequent inhibition of MMP-2 and cell migration.

*The chemical inhibitors and siRNAs showed no effects on cell migration, TIMP-3 mRNA, MMP-2 mRNA and miR-101 expression*

To investigate whether the inhibitors of c-Src, MEK, and ERK or their specific siRNA possess the inhibitory effects on cell migration, TIMP-3 mRNA, MMP-2 mRNA, and miR-101 in absence of S1P, these inhibitors or siRNA were applied. Results showed that either the abilities of cell migration or the TIMP-3 mRNA, MMP-2 mRNA, and miR-101 expression were not inhibited by all of the inhibitors or siRNA stimulation in JJ012 and SW1353 cells (Supplementary Figure 3A~3D).

**References**

Bartke, N., Hannun, Y.A., 2009. Bioactive sphingolipids: metabolism and function. J Lipid Res 50 Suppl, S91-96.

Sharma, L., Prakash, H., 2017. Sphingolipids Are Dual Specific Drug Targets for the Management of Pulmonary Infections: Perspective. Front Immunol 8, 378.

**Supplementary Figure Legends**

**Supplementary Figure 1. The TIMP-3 and MMP-2 expression were not regulated by other sphingolipid metabolites in human chondrosarcoma cells.**

Starved JJ012 and SW1353 cells were incubation with 2.5, 5, and 10 μM of S1P (A) or ceramide (B) for 24 hours, and the cell viability was then determined by MTT analysis. Starved JJ012 and SW1353 cells were incubation with 2.5, 5, and 10 μM of S1P, sphingosine, or ceramide for 24 hours, and TIMP-3 (C) and MMP-2 (D) mRNA expressions were analyzed through real-time PCR. Starved JJ012 and SW1353 cells were preincubation with 25 μM of fumonisin b1 for 1 hour, and followed by treatment with 10 μM of S1P for another 24 hours, the TIMP-3 (E) and MMP-2 (F) mRNA expressions were then determined by real-time PCR. Cells without treatments were used as a control (set to 1.0 or 100), and data were shown as multiples of that. Results are shown as the mean±SEM (n≧3). * *p* < 0.05 when compared to untreated control.

**Supplementary Figure 2. The S1P-inhibited human chondrosarcoma cell migration is not mediated through p38- and JNK-dependent pathway.**

(A) Starved JJ012 cells were incubated with 10 μM of S1P for 10, 15, 30, 60, and 120 min, and the phosphorylation of p38 and JNK were examined by immunoblotting using anti-phospho p38 and JNK antibodies, and followed by re-probing against to total p38, JNK, and β-actin to show the equal loading amounts. Starved JJ012 and SW1353 cells were preincubation with 10 μM of SB203580 or SP60012 for 1 hour, and followed by treatment with 10 μM of S1P for another 24 hours, the TIMP-3 (B) and MMP-2 (C) mRNA expressions or cell migratory ability (D) were then determined by real-time PCR and transwell analyses, respectively. Cells without treatments were used as a control (set to 1.0 or 100), and data were shown as multiples of that. Results are shown as the mean±SEM (n≧3). * *p* < 0.05 when compared to untreated control; ^#^ *p* < 0.05 when compared to S1P-treated group.

**Supplementary Figure 3. The cell migration, TIMP-3 mRNA, MMP-2 mRNA, and miR-101 expression were not regulated by chemical inhibitor or their siRNA stimulation in JJ012 and SW1353 cells.**

JJ012 (open bar) and SW1353 (closed bar) cells were treated with vehicle control or 10 μM of PP2, PD98059, and U0126, as well as the siRNA of c-Src, MEK, and ERK for 24 h, and the expressions of miR-101 (A), TIMP-3 mRNA (B), MMP-2 mRNA (C), or their abilities of migration (D) were measured by the real-time PCR analysis and transwell assay, respectively. Cells treated with vehicle control or transfected with scramble siRNA were used as a control (set to 100 or 1.0, respectively), and data were shown as multiples of that.
